# Analysis of pattern, time and risk factors influencing recurrence in triple-negative breast cancer patients

**DOI:** 10.1007/s12032-012-0388-4

**Published:** 2013-01-05

**Authors:** Katarzyna Pogoda, Anna Niwińska, Magdalena Murawska, Tadeusz Pieńkowski

**Affiliations:** 1 Department of Chemotherapy, Maria Sklodowska-Curie Memorial Cancer Center and Institute of Oncology, Warsaw, Poland; 2 Department of Breast Cancer and Reconstructive Surgery, Maria Sklodowska-Curie Memorial Cancer Center and Institute of Oncology, Warsaw, Poland; 3 Department of Biostatistics, Erasmus University Medical Center, Rotterdam, The Netherlands; 4 Department of Oncology, Postgraduate Medical Center, Warsaw, Poland

**Keywords:** Brain metastases, Metastatic breast cancer, Prognostic factor, Recurrence pattern, Triple-negative breast cancer, Tumor size

## Abstract

The aim of the study was to assess the rate, pattern, and time of recurrence in patients with triple-negative breast cancer (TNBC) and to evaluate factors influencing recurrence and overall survival in this group of patients. Out of 2,534 consecutive breast cancer patients diagnosed between January 2005 and December 2006, 228 (9 %) were TNBC (ER/PR/HER2-negative). The clinicopathological characteristics were determined using descriptive statistics. The overall survival (OS) and disease-free survival (DFS) were calculated using the Kaplan–Meier method. The univariate and multivariate analyses were developed to identify factors influencing recurrence and survival in TNBC patients. After 6 years of observation, metastatic disease occurred in 35 % of all TNBC patients: 15 % in the brain, 14 % in the lungs, 11 % in the bones, 8 % in the liver, and 14 % had locoregional relapse. The highest risk of recurrence was during the first 3 years after primary treatment, and then, during the next 2 years of observation, it did not change. 6-year DFS and OS were 68 and 62 %, respectively. Factors influencing recurrence were tumor size and systemic adjuvant chemotherapy, while factors influencing overall survival were tumor size, nodal status, adjuvant/neoadjuvant treatment, and metastases in the brain, liver, and bones. Characteristic pattern of recurrence in time was revealed. The tumor size was responsible for recurrence despite lack of involvement of lymph nodes. Aggressive adjuvant/neoadjuvant treatment ordered in all clinical stages of TNBC (including N0) was factor responsible for avoiding local and distant relapse and prolonging overall survival.

## Introduction

According to ASCO guideline and the latest St Gallen consensus, triple-negative breast cancer (TNBC) occurs only if there is no expression of estrogen receptor (ER-negative), progesterone receptor (PR-negative), and there is neither expression nor amplification of human epidermal growth factor receptor 2 (HER2-negative) in a tumor [1, 2]. Apart from these clinicopatological markers and classical classification of breast cancer subtypes, there are molecular gene tests, which allow dividing TNBC into two main subtypes—more common basal-like and claudin-low [3]. Still, molecular classification has still no influence over clinical management. TNBC accounts for about 9–21 % of all breast cancers including patients with stage I–IV breast cancer [4–8]. In the past, this rate was higher because it included cases with ER/PR less than 10 %.

TNBC patients have poorer outcomes compared with other cancer subtypes [7–14]. They are at higher risk of early recurrence, mainly in the lungs, brain, and soft tissue [4, 6, 13–18]. The highest risk of relapse is between the first and third year after primary treatment. In cases of recurrence, the survival is shorter than in non-TNBC patients [9, 13, 14, 19, 20]. However, TNBC is more sensitive to chemotherapy. The rate of pathological complete remission (pCR) after neoadjuvant chemotherapy is higher than in other breast cancer subtypes [11, 13, 19]. On the other hand, methods of the treatment in this group of patients are still limited in clinical practice because of the lack of molecular targets. The adjuvant treatment is usually recommended in TNBC and should include anthracyclines, taxanes, and an alkylating agent [2, 21].

The aim of this study was to analyze recurrence pattern in order to determine the prognostic factors of recurrence and overall survival in a group of consecutive 228 TNBC patients treated at Cancer Center and Institute of Oncology in Warsaw, Poland, between the years 2005 and 2006.

## Patients and methods

### Patients

Medical records of 2,534 consecutive patients with newly diagnosed breast cancer between January 2005 and December 2006 were reviewed. We decided to analyze this group of patients because of similar management, and the fact that most of recurrence in TNBC patients occurs in the first 5 years after primary treatment. There were 228 TNBC patients (9 % out of all breast cancers) according to the latest recommendations [1, 2], therefore, without expression of ER, PR, and HER2 receptors. We excluded 23 patients who were in the past also classified as TNBC (with expression of ER/PR receptors less than 10 %). The status of ER, PR, and HER2 was determined based on the biopsy of primary tumor. Staining was performed using primary antibodies against ER (Clone 6F11, Novocastra), PR (Clone 16, Novocastra), and HER2 (Polyclonal HercepTest, DAKO). If HER2 was 2+ by immunohistochemistry (IHC), fluorescence *in situ* hybridization (FISH; HER2 DNA Probe Kit, Vysis) was performed additionally, and if the result was only negative, patients were included to our analysis. Patients were observed until October 2011.

### Statistical method

The clinicopathological characteristics were determined using descriptive statistics.

Disease-free survival was defined as the time from diagnosis of TNBC to first locoregional or distant recurrence. Overall survival was the time from TNBC diagnosis to death. DFS and OS curves were calculated using the Kaplan–Meier method. Recurrence rates were presented by cumulative hazard rates and annual hazard rates. A *p* value <0.05 was considered significant.

The univariate and multivariate analyses were developed to identify factors influencing recurrence and overall survival in TNBC patients. The following factors were analyzed in Cox model: age at diagnosis (<55 vs. ≥50), primary tumor extension (T2 vs. T1; T3 vs. T1; T4 vs. T1), lymph node involvement (N1 vs. N0; N2 vs. N0; N3 vs. N0), histological cancer type (ductal vs. lobular; ductal vs. medullar, apocrinal, papillary), result of HER2 in IHC staining (HER2 1+ vs. HER2 2+), adjuvant/neoadjuvant chemotherapy (yes vs. no).

## Results

### Patients

The median age of patients at diagnosis was 54.5 years (range 24–86) (Table 1). Majority of patients were diagnosed with stage II or III breast cancer (47 and 34 %, respectively), and only 9 patients (4 %) had an evidence of metastases at initial diagnosis. 126 patients (55 %) had positive axillary lymph nodes at presentation. The most common histological type was ductal cancer (81 %). 71 patients (31 %) were treated with neoadjuvant chemotherapy due to locally advanced breast cancer—almost half of them with 4 cycles AT (doxorubicin plus docetaxel) followed by 4 cycles of CMF (cyclophosphamide plus methotrexate plus fluorouracil)—and two-thirds of these patients received the taxane-containing regimens. Of note, pCR rate in all types of neoadjuvant chemotherapy was only 9.9 % (7 patients). There was no difference in overall survival regardless of the type of neoadjuvant chemotherapy given. 90 % of patients received surgery, and in this group, mastectomy was the most common type of surgery (85 %). The rate of breast-conserving surgery was only 15 % because many patients had stage III cancer. Almost half of all patients received adjuvant chemotherapy—AC (doxorubicin plus cyclophosphamide) was the most common regimen; taxanes were used only in 12 % of patients in this group.

**Table 1:** Characteristics of 228 triple-negative breast cancer patients

Patient and tumor characteristics	No. of patients	%
Age at initial diagnosis, years	54.5	
Median		
Range	24–86	
Initial clinical TNM stage		
I	34	15
II	108	47
III	77	34
IV	9	4
Histological cancer type		
Ductal invasive	153	81
Lobular invasive	13	7
Medullar, apocrinal, papillary, mucinous, planoepitheliale, neuroendocrine invasive	23	12
Cancer cells or invasive cancer after chemotherapy	39	–
Systemic neoadjuvant treatment		
Yes	71	31
No	157	69
Type of neoadjuvant chemotherapy—regimens		
AT + CMF	32/71	45
AT	15/71	21
With anthracycline	20/71	28
CMF	2/71	3
Other	2/71	3
Surgical treatment		
Mastectomy	175	77
Breast conservation	31	13.50
No	22	9.50
Systemic adjuvant therapy		
Yes	118	49
No	110	51
Type of adjuvant chemotherapy		
AC	79/118	67
CMF	15/118	12.50
A + T	14/118	12
FEC or FAC	7/118	6
Other	3/118	2.50
Adjuvant radiotherapy		
Yes	111	51
No	117	49
Recurrence		
Yes	79	35
No	149	65
Site of initial recurrence		
Lung	29	13
Locoregional recurrence	28	12
Brain	19	8
Bone	18	8
Liver	18	8
Other	5	2
Site of initial and subsequent metastases		
Brain	34	15
Lung	33	14
Bone	26	11
Liver	19	8
Locoregional recurrence	31	14
Other	13	6
Systemic therapy after recurrence		
Yes	54/79	68
No	25/79	32
Type of systemic therapy after recurrence*		
Chemotherapy	53/79	67
Hormonal therapy	7/79	9
Targeted therapy	10/79	13
Type of chemotherapy—schedules with*		
Anthracycline	20/53	38
Taxane	27/53	51
Vinorelbine	24/53	45
Capecitabine	13/53	25
Platinum	8/53	15
Other	8/53	15
Type of targeted therapy		
Bevacizumab	9/10	90
Trastuzumab	1/10	10
Number of lines of chemotherapy in metastatic setting		
0	26/79	33
1	23/79	29
2	18/79	23
3	12/79	15
Second breast cancer	5	2
Other primary cancer	12	5

*Some patients received different types of systemic therapy

One-third of patients with metastatic disease did not receive systemic therapy, mainly due to their poor functional status. First-, second-, and third-line chemotherapy were used in 23 patients (29 % of all patients with metastases), 18 patients (23 %), and 12 patients (15 %), respectively. A few patients received hormonal therapy—only in cases, where ER/PR conversion occurred in metastases. Bevacizumab was used in 9 patients, only in addition to first-line chemotherapy (in 1 case, bevacizumab was continued with second-line chemotherapy). One patient received trastuzumab in metastatic setting because HER2 conversion occurred.

### Recurrence

During the 6 years of observation, the metastatic disease occurred in one-third of all TNBC patients (including these 9 patients initially with stage IV disease): 15 % in the brain, 14 % in the lungs, 11 % in the bones, 8 % in the liver, and 14 % patients had locoregional relapse (Table 1)—majority of these patients had metastases in different sites. The most common site of the first recurrence was lungs. Interestingly, almost half of all brain metastases occurred in patients with lung involvement—in this group of patients, lung metastases were diagnosed before or concurrently with brain metastases apart from 1 patient with prior brain disease.

**Fig. 1:**
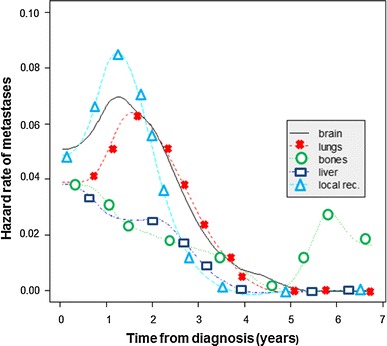
Risk of recurrence in different sites in triple-negative breast cancer patients

**Fig. 2:**
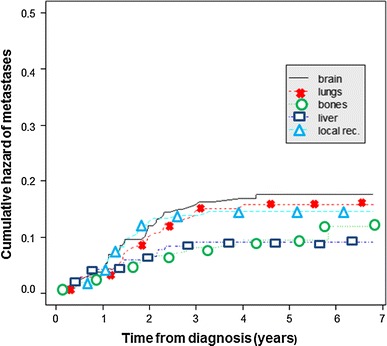
Cumulative hazard rates depending on the site of metastases

The highest risk of recurrence was during the first 3 years after primary treatment, and then, during the next 2 years of observation, it did not change significantly (plateau after 3 years; Figs. 1, 2). In the study population, the risk of local relapse and metastases to the brain and lungs peaked in second year and then declined significantly, whereas the risk of metastases to the liver and bones was also the highest in the first 2–3 years but then fell slightly. Furthermore, 5 years after initial diagnosis new metastases occurred only in bones. However, longer follow-up is needed to complete and verify these results.

Cumulative hazards of metastases are presented in Fig. 2 and Table 2. The risk of new metastases after 3 years from primary treatment was very low (<1 %/year in every site).

**Table 2:** Cumulative hazard of metastases

Site of metastases	After 1 year (%)	After 2 years (%)	After 3 years (%)	After 5 years (%)
Brain	4.3	12	16	17.6
Lungs	3.8	10.7	15.1	15.7
Liver	4.2	6.5	9.1	9.1
Bones	4.3	6.7	8.1	9.4
Local recurrence	3.9	13.3	13.9	14.6

### Survivals

**Fig. 3:**
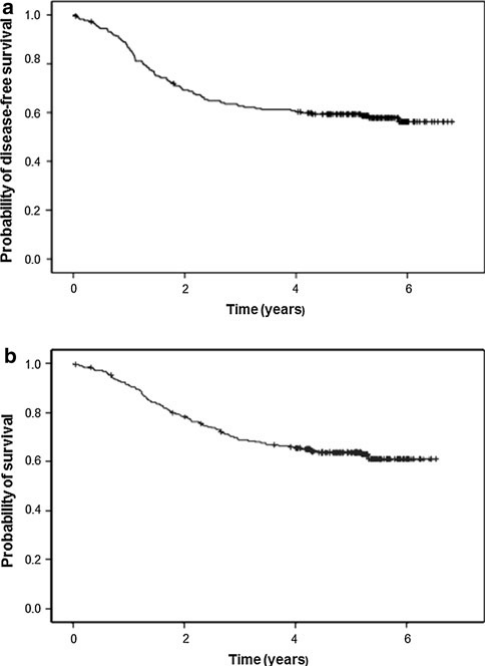
**a** Disease-free survival (DFS) and **b** overall survival (OS) in triple-negative breast cancer patients

Median DFS and OS were not reached at the time of analysis, and 6-year DFS and OS were 68 and 62 %, respectively (Fig. 3). Less than half of all patients experienced recurrence or died, so there was only possibility to estimate the means of DFS and OS (4.4 and 5 years, respectively).

88 patients (39 %) died during 6-year observation. In 19 patients, cause of death was not associated with breast cancer. 12 patients (5 %) developed other primary cancer (pancreatic cancer, colon cancer, and ovarian cancer—2 cases; lung cancer, melanoma, kidney cancer, endometrial cancer, cancer of the ampulla of Vater, and skin cancer—1 case) and half of them (6 patients) died; only 2 of these patients had also metastatic breast cancer, but it did not cause their death.

The survival after recurrence differed depending on the site of metastases (Table 3) and was the shortest in the group of patients with liver metastases (3.5 months). The longest survival occurred in patients with lungs metastases and with local relapse (9.8 and 9 months, respectively). The median survival from the detection of brain metastases was 6.3 months.

**Table 3:** Survival after recurrence depending on the site of metastases

Site of metastases	No. of patients	Median (months)	95 % CI (months)
Brain	34	6.3	4.9–7.7
Lungs	33	9.8	1.7–17.8
Local recurrence	31	9	7.5–10.6
Bones	26	5.5	2.7–8.4
Liver	19	3.5	0–7.7

### Prognostic factors

#### Factors influencing disease-free survival

In the univariate analysis, tumor size, nodal status, and adjuvant/neoadjuvant chemotherapy were found to have significant impact on DFS. Additionally, there was no difference in DFS in patients with HER2 1+ or 2+ in IHC staining. Patients’ age also did not influence DFS. However, in the multivariate analysis, only tumor size and systemic adjuvant chemotherapy were significant for DFS (Table 4).

**Table 4:** Factors influencing disease-free survival (DFS) and overall survival (OS)—multivariate analysis, final model

Factor	Hazard ratio	95 % confidence interval	Log-rank *p*
DFS			
T4	15.99	3.55–71.95	0.000
T3	16.39	3.53–76.11	0.000
T2	8.13	1.95–33.77	0.004
Adjuvant/neoadjuvant chemotherapy vs. no chemotherapy	0.38	0.20–0.73	0.004
Chemotherapy stage III vs. other stages	3.18	1.48–6.84	0.003
OS			
Brain metastases	2.00	1.20–3.33	0.008
Bone metastases	2.13	1.25–3.63	0.005
Liver metastases	2.06	1.01–4.19	0.047
Local recurrence	2.34	1.39–3.96	0.001
Adjuvant/neoadjuvant chemotherapy vs. no chemotherapy	0.40	0.24–0.67	0.000
T4	11.56	2.63–50.93	0.001
T3	8.21	1.71–39.29	0.008
T2	4.47	1.05–19.00	0.042
N3	3.95	1.27–12.27	0.018
N2	2.59	1.32–5.09	0.006
N1	1.77	1.03–3.07	0.040

#### Factors influencing overall survival

The same factors for DFS were analyzed as for OS. Additionally, the development of metastases in different sites was parsed. Similar results were found—tumor size, nodal status, and adjuvant/neoadjuvant chemotherapy were significant in univariate analysis while patients’ age and HER2 result in IHC (1+ vs. 2+) was not. The risk of death was higher in cases with evidence of metastases in every location. Finally, in multivariate analysis tumor size, nodal status, adjuvant/neoadjuvant treatment, and metastases to the brain, liver, and bones were factors influencing OS.

## Discussion

The old term for TNBC included tumors with low expression of hormonal receptors (ER/PR < 10 %). There are a lot of publications with data referring to this old classification making the rate of TNBC tumors to be higher in the past than nowadays. In our study, this rate was 9 % and is comparable to other studies (9–21 %) [4–8]. The rate of TNBC patients was in some studies even higher, but they assessed other groups of TNBC patients (e.g. African American patients, only neoadjuvant setting or patients with stage I–III breast cancer) [12, 13, 22].

The frequency of nodal involvement at diagnosis in TNBC patients differs in studies with conflicting results. Lin et al. demonstrated recently that TNBC tumors were less likely to be lymph node-positive, and a similar outcome was reported in other studies (positive lymph nodes: 38 and 41 %, respectively) [14, 22]. Contrary, in our study, 55 % of TNBC patients had lymph node involvement, which is consistent with the frequency of 54.4 % reported in other study [9]. Nodal status issue remains unresolved.

Neoadjuvant chemotherapy consisting of anthracyclines and taxanes has been widely accepted as standard therapy of locally advanced breast cancer [21]. Patients with TNBC have increased pCR rates compared with non-TNBC. This rate was 29 % in patients who received neoadjuvant anthracycline-based chemotherapy and 38 % after anthracycline and taxane combined treatment [11, 19]. In our study, two-thirds of patients received anthracycline-taxane neoadjuvant chemotherapy and only 15 % of them achieved pCR—recurrence occurred in almost half of patients in this small group. However, such result is doubtful because there were only 7 patients who achieved pCR (all of them received chemotherapy containing taxanes); the result was lower than normal. In addition, it was reported that if pCR was achieved, patients with TNBC and non-TNBC would have similar survival [13, 19].

TNBC has a characteristic pattern of recurrence. Dent et al. [9] reported that in their study the risk of recurrence rose sharply from the date of diagnosis, peaked at 1–3 year interval and then dropped quickly. Similarly, in another study, the risk of relapse was strongly time-dependent and dramatically higher for TNBC patients during the first 3 years after diagnosis [13]. The same phenomenon was observed in our study. Additionally, we determined the sequence of metastases development in different sites. Local relapse and metastases to the brain and visceral organs occurred in the first 3 years, but the risk of bone metastases declined from diagnosis and then rose slightly again after 5 years of observation (Fig. 1). The brain and lungs were the most common sites of recurrence which is in partial agreement with the results from other studies. Lin et al. [16] analyzed the sites of distant recurrence in 116 metastatic TNBC patients and reported that the majority of metastases were in lungs and liver. The brain was the third most common site of recurrence. However, in recently published study, TNBC tumors were associated with a greater risk of brain and lung metastases [14]. In contrast, in other studies, the rates of local relapse or bone metastases were the highest [4, 23]. The frequency of liver metastases in our study was low; this result was consistent with the observation from a previous study [24]. According to initial staging in our study, the recurrences were 1 % (1 patient), 36 % (25 patients), and 63 % (44 patients), respectively, for patients with stages I, II, and III breast cancer. These data were similar to results described by Alarcon-Rozas et al. [25] (7.5, 32.5, and 60 %, respectively).

After a 6 year follow-up, DFS and OS in our study were 68 and 62 %, respectively. These results accord with a previous study which did not include TNBC patients with stage IV; 5-year DFS and OS were 68.2 and 74.5 %, respectively [26]. However, in the above-mentioned Kaplan et al. [7] study, 5-year relapse-free survival (RFS) and OS in TNBC patients were 84 and 81 %, respectively. This result may be dependent on the difference in the staging of breast cancer between studies—in our study, majority of patients had more advanced disease (more patients with stage III and less with stage I), whereas nearly 80 % of patients in Kaplan study presented with stage I or II.

In a recent retrospective study, TNBC metastatic patients were divided into two subgroups by RFS [27]. The analysis showed that patients with RFS ≥ 3 years had better outcomes—higher disease control rate (DCR), longer progression-free survival (PFS) to first-line palliative chemotherapy, and longer OS than those with RFS < 3 years (DCR 55 vs. 77 %, *p* = 0.022; median PFS 3.6 vs. 7.7 months, *p* = 0.0001; median OS 17.4 vs. 42.0 months, *p* = 0.0003). In our study, 155 patients (71 %) had RFS ≥ 3 years. Only 7 patients experienced recurrence after 3 years from primary diagnosis and 3 of them died. On the other hand, almost all patients with RFS < 3 years passed away (59 of 63 patients).

Furthermore, patients with brain metastases have poor outcomes. The median survival from brain metastases has been reported to be between 2.9 and 4.9 months, compared to 6.3 months in our study [6, 15–18, 28]. Brain metastases were the first site of recurrence in 19 patients (8.3 %) (in some of them metastases occurred concurrently in other sites). None of the patients developed brain metastases at the diagnosis of breast cancer. On the other hand, in a recent study, the incidence of brain metastases as the first site of recurrence in TNBC patients initially at stage I–III was 4.7 % [29]. The incidence of brain metastases as the first site of relapse in the in the above-mentioned study led by Park was much more common in patients with shorter RFS than in those with longer RFS (16 vs. 3 %, *p* = 0.047) [27]. Similar results were found in our study—in the same group of patients (metastatic TNBC, initially presented with stages I–III), brain metastases were more likely to develop in patients with RFS < 3 years then with RFS ≥ 3 years (24 and 3 %, respectively).

The most relevant factor responsible for survival in this study was tumor size. The hazard ratio (HR) of recurrence in patients with a tumor >5 cm was 16 times higher than among patients with a tumor <2 cm. A hazard for death was also elevated in patients with large tumors (HR = 8.21 in tumors >5 cm). These results were in agreement with a previous report where the tumor size was the most important prognostic factor in TNBC patients [5]. Even small, node-negative (T1N0) TNBC tumors appear to have a higher recurrence rate, which was documented in some studies as well [30, 31]. According to these observations, TNBC patients should be given more aggressive treatment, even if they are in a low-risk category. Apart from tumor size, the patients’ prognosis in our and other studies depended also on nodal status [8, 32]. In a recent study, survival did not differ among patients with N1, N2, and N3 [33]. In contrast, some analysis showed that TNM staging was not sufficient for predicting therapeutic outcome for TNBC patients due to the different biology of this breast cancer subtype [34].

It is known that TNBC affects younger women. However, one question that should be addressed is whether younger age is associated with poor prognosis in this group of patients. The results of studies are ambiguous. Kassam et al. [35] reported that TNBC patients <50 years had worse outcome. In other study, age was not related to prognosis [8]. On the other hand, Ovcaricek et al. [26] showed that age >65 years was an independent prognostic factor for DFS and that the risk of recurrence was 1.79-fold higher in older patients than in younger patients. Interestingly, in the univariate analysis of our study, older patients had shorter survival (age > 65 vs. < 65 years, *p* = 0.036), but this result lost its independent prognostic value in the multivariate analysis. Only 55 % of patients older than 65 years received adjuvant/neoadjuvant chemotherapy (among stage I–III TNBC), whereas this rate in younger patients was 91 %. This can explain our founding at least partially.

To the best of our knowledge, this is the largest study evaluating TNBC in Poland. Recurrence rates for different sites were presented by cumulative hazard rates and annual hazard rates. However, longer time of observation is necessary to complete and verify these results, especially in terms of recurrence in different sites.

Patients and oncologists always have a dilemma on how to cope with this aggressive disease. For patients, the diagnosis of breast cancer is fearsome; so when they know additionally that they suffer from TNBC—a subtype with poor outcomes—this situation is often more stressful. We recommend that this group of patients should be offered to participate in clinical trials with novel agents.

## Conclusions

TNBC patients have a unique pattern of relapse, which occurs mostly in the first 3 years following diagnosis. The most common sites of recurrence were brain and lungs. Tumor size was independent prognostic factor for prognosis, and this feature should be mainly considered in the management of TNBC patients. This group of patients should receive aggressive adjuvant therapy to prevent early recurrence or death. Further prospective clinical trials are needed to identify the most efficient therapy in order to improve survival outcomes.
